# Comparison of Testicular Workup for Ischemia and Suspected Torsion (TWIST), Testicular Torsion (TT Lim), and Boettcher Alert (BAL) Scoring Systems for the Diagnosis of Testicular Torsion in 50 Surgical Cases

**DOI:** 10.7759/cureus.98125

**Published:** 2025-11-30

**Authors:** Shohei Tobu, Yukako Yamaguchi, Minika Yukimoto, Akihiro Maeda, Shuhei Kusano, Yuka Kakinoki, Hiroaki Kakinoki, Maki Kawasaki, Mitsuru Noguchi

**Affiliations:** 1 Department of Urology, Saga University, Faculty of Medicine, Saga, JPN; 2 Department of Urology, Saga University Faculty of Medicine, Saga, JPN

**Keywords:** acute scrotum, bal score, testicular torsion, tt (lim) score, twist score

## Abstract

Objective

The objective of the study was to compare the diagnostic accuracy of three clinical scoring systems (Testicular Workup for Ischemia and Suspected Torsion (TWIST), Testicular Torsion Lim (TT developed by Lim), and Boettcher Alert (BAL) scoring systems) for testicular torsion (TT) in patients presenting with acute scrotum.

Methods

This retrospective study included 50 patients who underwent emergency scrotal exploration at the Saga University Hospital between January 2010 and December 2023. The TWIST, TT (Lim), and BAL scores were calculated from medical records, and their diagnostic performance was assessed using sensitivity, specificity, positive predictive value (PPV), and negative predictive value (NPV). The study protocol was approved by the Institutional Review Board of Saga University.

Results

Out of 50 patients, 34 (68%) were diagnosed with TT. The TWIST score showed 88.2% sensitivity, 43.8% specificity, 90.9% PPV, and 87.5% NPV. The TT (Lim) score demonstrated 85.3% sensitivity, 18.8% specificity, 96.7% PPV, and 100% NPV. The BAL score showed the highest sensitivity (94.1%) but lower specificity (37.5%), with a PPV of 76.2% and an NPV of 75.0%. All scores showed statistically significant associations with the TT diagnosis (p<0.01).

Conclusions

The TWIST, TT (Lim), and BAL scores have unique advantages in evaluating an acute scrotum. BAL may be most suitable for initial screening because of its high sensitivity, whereas TWIST may aid surgical decision-making. The TT score may be useful in ruling out torsion in selected low-risk cases, although validation in larger cohorts is required.

## Introduction

Acute scrotum is a relatively common presentation in emergency medicine and requires a rapid and accurate differential diagnosis. The diagnosis of acute scrotal pain and swelling in children is often challenging and time-sensitive. Knight and Vassy emphasized that early surgical exploration remains the diagnostic and therapeutic procedure of choice in pediatric acute scrotum [1]. In particular, testicular torsion (TT) is a surgical emergency requiring early detorsion, and delayed treatment can result in irreversible testicular ischemia and eventual orchiectomy. Rampaul and Hosking further demonstrated that late presentation is a major cause of testicular loss, as all patients who required orchidectomy presented more than six hours after symptom onset [2].

Acute scrotum is a relatively common presentation in emergency medicine and requires a rapid and accurate differential diagnosis. In particular, TT is a surgical emergency requiring early detorsion, and delayed treatment can result in irreversible testicular ischemia and eventual orchiectomy. TT frequently occurs in children and adolescents, accounting for approximately 10-15% of acute scrotal cases seen in emergency departments [3].

In Japan, the incidence of TT in individuals under 21 years of age is reported to be 15.1 per 100,000 population, with an orchiectomy rate of approximately 7.1% [4]. These statistics underscore the importance of early differentiation and treatment in patients with acute scrotum.

Among the various clinical scoring systems proposed for the evaluation of TT, the Testicular Workup for Ischemia and Suspected Torsion (TWIST) score is one of the most widely used [5]. It includes five components: testicular swelling, hardness, high-riding testis, absent cremasteric reflex, and nausea/vomiting, with a total possible score of 7. A score ≥5 indicates a high risk. Meta-analyses have demonstrated a sensitivity of 0.97, a specificity of 0.97, a negative predictive value of 0.71, and a positive predictive value of 90% [6,7].

Lim et al. proposed a new scoring system in 2020 [8]. The TT (Lim) score assigned two points for nausea/vomiting and high-riding testis, one point for testicular hardness, and one point for patients <1 year or ≥10 years of age. Designed for intuitive use by non-specialists, the TT (Lim) score incorporates both physical examination and age-based criteria. Their study categorized scores of 6 or higher as the high-risk group, in which the likelihood of testicular torsion was markedly increased [8].

Boettcher developed the Boettcher Alert (BAL) score, which targets the pediatric population. This score includes four items: symptom onset within 24 hours, nausea/vomiting, high-riding testis, and absent cremasteric reflex. A score of ≥2 indicates high risk. The prediction study detected all 48 TT cases in 460 patients using this score [9].

These scoring systems are useful tools to complement color Doppler ultrasound (CDUS) in early emergency triage. However, their diagnostic accuracies differ, and appropriate score selection is necessary depending on the clinical setting.

This retrospective study aimed to compare the diagnostic accuracies of the TWIST, TT (Lim), and BAL scores in 50 patients who underwent emergency surgery for acute scrotum at our institution. We evaluated the sensitivity, specificity, PPV, and NPV of each score to identify their clinical utility and limitations and to develop an optimized diagnostic algorithm for TT.

Although TWIST, TT (Lim), and BAL are all validated tools, they differ in intended users and criteria: TWIST balances five classic clinical signs, TT (Lim) adds age weighting to simplify use by non-specialists, and BAL prioritizes rapid screening in pediatrics. Head-to-head evaluations across the same clinical cohort remain limited, particularly in Asian settings. Moreover, many published accuracies come from mixed emergency-department populations, whereas clinicians often need to decide immediately before or during surgical triage. We therefore compared these three scores within a uniform, surgically explored cohort to clarify their complementary roles in real-world preoperative decision-making. We also explicitly addressed how surgical-only sampling influences prevalence, sensitivity, and PPV, and we position our findings accordingly for cautious, context-specific use. 

This study was previously presented at the 112th Annual Meeting of the Japanese Urological Association, held in Fukuoka, Japan, on April 17-19, 2025.

## Materials and methods

This retrospective observational study included 50 patients who underwent emergency scrotal exploration for acute scrotum at the Department of Urology, Saga University Hospital, between January 2010 and December 2023. All patients had a definitive intraoperative diagnosis of either TT or a non-TT disease. Patient ages ranged from one to 42 years, with a mean of 15.6±10.7 years.

Case identification and screening

We retrospectively screened all emergency presentations with acute scrotal pain or swelling at our institution from January 2010 to December 2023. Of 216 encounters, 105 had sufficient clinical documentation to reconstruct all variables required for TWIST, TT (Lim), and BAL. Among these, 50 underwent urgent scrotal exploration based on clinical judgment (with or without CDUS), and 50 surgically explored cases comprised the analytic cohort. This approach allowed to appraise potential selection bias introduced by record completeness and by restricting to surgical cases.

Inclusion and exclusion criteria

Patients who underwent emergency scrotal exploration for an acute scrotum were eligible. Inclusion criteria included patients with acute scrotal pain or swelling within 48 hours of symptom onset and complete medical records allowing calculation of the TWIST, TT (Lim), and BAL scores. Exclusion criteria applied for patients with chronic or recurrent scrotal pain for more than 48 hours of duration, prior scrotal or inguinal surgery, incomplete medical data, and uncertain intraoperative diagnosis.

Scoring systems

The original publications describing each scoring system are found in the references: TWIST score [5], TT (Lim) score [8], BAL score [9]. The TWIST score (maximum of 7 points), the TT (Lim) score (maximum of 9 points), and the BAL score (maximum of 4 points) were retrospectively calculated based on medical records, according to the criteria described in their original publications. For analysis, patients were categorized into risk groups according to the definitions established in the original studies. TWIST scores of 0-2 were defined as low risk, 3-4 as intermediate risk, and 5-7 as high risk. TT (Lim) scores of 0-1 were defined as low risk, 2-5 as intermediate risk, and 6-9 as high risk. BAL scores of 0-1 were defined as low risk, and 2-4 as high risk. 

Blinding of score abstraction 

To mitigate retrospective confirmation bias, two reviewers (authors AM, YY) independently abstracted all score components from ED triage notes and physical examination records while blinded to the intraoperative diagnosis and postoperative course; discrepancies were adjudicated by a third reviewer (author ST).

All data were collected retrospectively and anonymized prior to analysis. 

Data analysis

Continuous variables were expressed as mean ± standard deviation (SD), and categorical variables as numbers and percentages. Diagnostic performance, including sensitivity, specificity, positive predictive value (PPV), and negative predictive value (NPV), was calculated using standard formulas. The association between each score category and the presence of torsion was analyzed using Chi-square tests. Receiver operating characteristic (ROC) curves were constructed to evaluate discriminative ability. All analyses were performed using Ekuseru-Toukei 2015 (Social Survey Research Information Co., Tokyo, Japan), and p<0.05 was considered statistically significant.

## Results

A total of 50 patients who underwent emergency scrotal exploration were included in this study. Among them, 34 (68.0%) were diagnosed with TT, and 16 (32.0%) were diagnosed with non-TT conditions such as epididymitis. The mean age of the entire cohort was 15.6±10.7 years. The mean TWIST, TT (Lim), and BAL scores for all patients were 5.0±2.1, 5.2±1.9, and 2.8±1.1, respectively (see Table 1).

**Table 1:** Patient characteristics TT - testicular torsion; TWIST score - Testicular Workup for Ischemia and Suspected Torsion score; BAL score - Boettcher Alert score

Variable	Group	n (%) or mean±SD
Age (years)	-	15.6±10.7
Testicular torsion	Yes	34 (68.0%)
Testicular torsion	No	16 (32.0%)
Side (TT group only)	Left	22 (64.7%)
Side (TT group only)	Right	12 (35.3%)
TWIST score	-	5.0±2.1
TT (Lim) score	-	5.2±1.9
BAL score	-	2.8±1.1

Comparison of risk categories and torsion status

**Figure 1:**
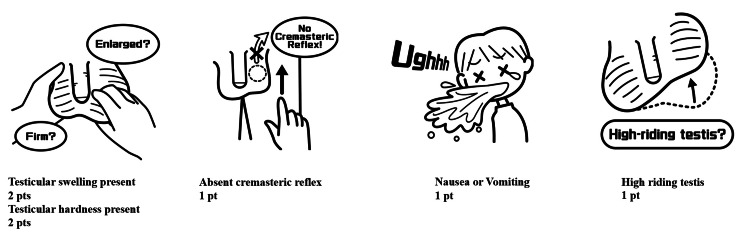
TWIST score components and conceptual illustrations This diagram illustrates the five clinical elements that comprise the TWIST score for evaluating testicular torsion in children. Each element was assigned a score based on its clinical relevance. The original concept was drawn by the author (ST) based on the descriptions provided in [5], and the final illustrations were professionally prepared by a medical illustrator commissioned by the authors (image credit: Masanao Amano). Conceptual illustration created by the authors based on the concept of the TWIST score [5]. This figure is an original creation and not a reproduction of the original work. TWIST score - Testicular Workup for Ischemia and Suspected Torsion score

**Figure 2:**
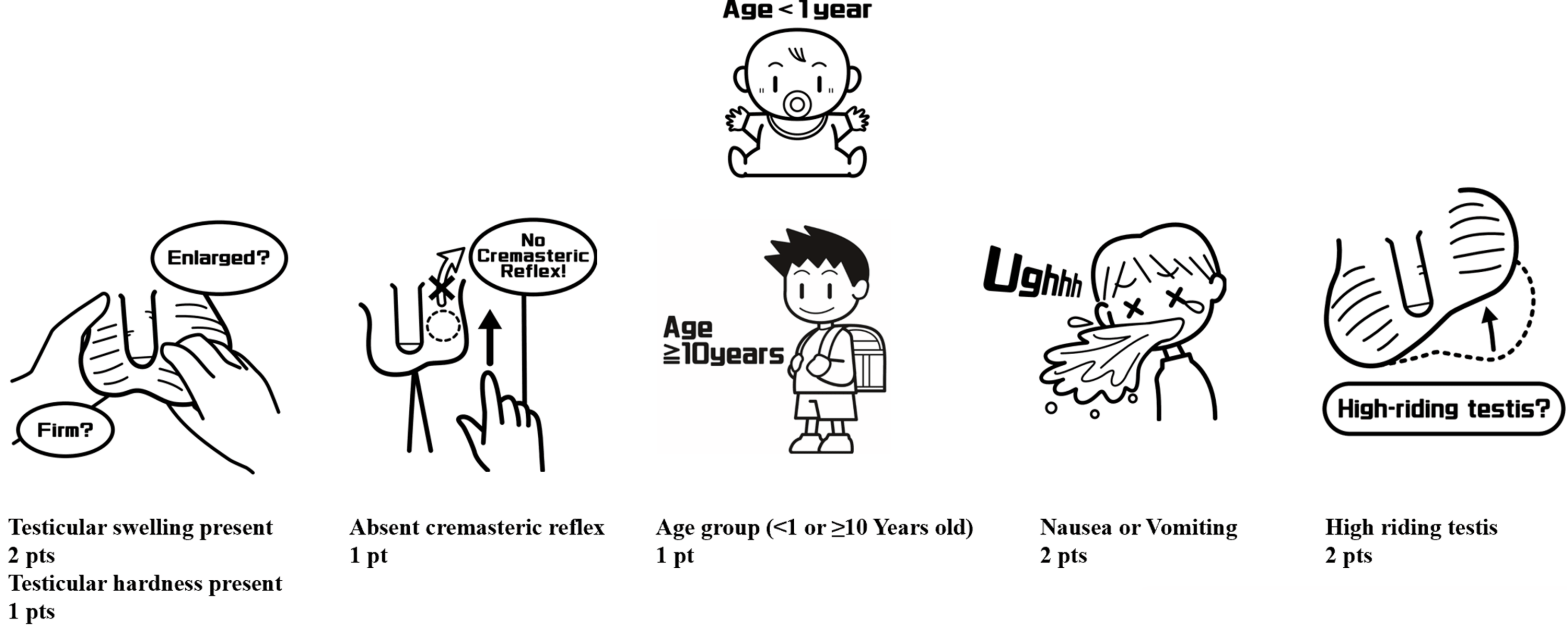
TT (Lim) score components and conceptual illustration This diagram illustrates the five clinical elements that compose the TT (Lim) score. Each element is assigned a score based on its clinical relevance, including age-based criteria. The original illustrations were drawn by the author (ST) based on the published descriptions [8], and the final versions were professionally prepared by a medical illustrator commissioned by the authors (image credit: Masanao Amano). Conceptual illustration created by the authors based on the concept of the TT (Lim) score [8]. This figure is an original creation and not a reproduction of the original work. TT - testicular torsion

**Figure 3:**
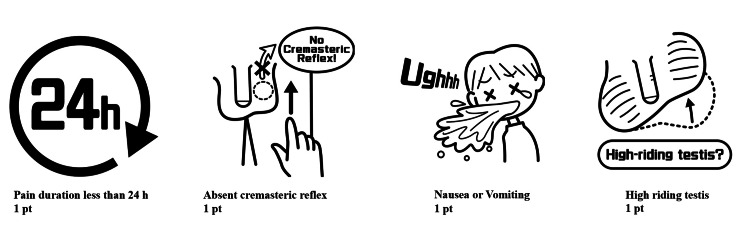
BAL score components and conceptual illustration This diagram illustrates the four clinical elements that compose the BAL score for evaluating testicular torsion in children. Each element is assigned a score based on its clinical relevance. The original illustrations were drawn by the author (ST) based on the published descriptions [9], and the final versions were professionally prepared by a medical illustrator commissioned by the authors (image credit: Masanao Amano). Conceptual illustration created by the authors based on the concept of the BAL score [9]. This figure is an original creation and not a reproduction of the original work. BAL score - Boettcher Alert score

The structure and clinical components of each scoring system are illustrated in Figures 1-3. In the TWIST scoring system (Figure 1), 30 of 33 patients classified as high risk (5-7 points) had TT, while 7 of 8 patients with low-risk scores (0-2 points) were non-TT cases. The Chi-square test showed a significant association between higher TWIST scores and TT (p<0.01). Similarly, in the TT (Lim) system (Figure 2), 29 of 30 patients in the high-risk category (6-9 points) had TT, and all three patients in the low-risk group (0-1 point) were non-TT cases. The Chi-square test again demonstrated a significant relationship between risk category and torsion status (p<0.01). For the BAL score (Figure 3), 32 of 42 patients in the high-risk category (2-4 points) were diagnosed with TT, and 6 of 8 low-risk patients were non-TT patients. These results also showed a significant association with TT (p<0.01).

Diagnostic accuracy of the scoring systems

The diagnostic performance of the three scoring systems is summarized in Table 2. The TWIST score showed a sensitivity of 88.2% and specificity of 43.8%, with a positive predictive value of 90.9% and a negative predictive value of 87.5%. The TT (Lim) score demonstrated a sensitivity of 85.3%, specificity of 18.8%, PPV of 96.7%, and NPV of 100%. The BAL score had the highest sensitivity (94.1%) but a moderate specificity (37.5%), with PPV and NPV of 76.2% and 75.0%, respectively. TWIST had the highest balance between sensitivity and specificity, with PPV and NPV also above 85%. Receiver operating characteristic (ROC) analysis indicated that both TWIST and TT (Lim) scores achieved an area under the curve (AUC) exceeding 0.85, while the BAL score showed moderate discriminative ability (AUC of 0.76). These results suggest that each scoring system has distinct strengths in different clinical contexts.

**Table 2:** Comparison of diagnostic performance of TWIST, TT (Lim), and BAL scores TT - testicular torsion; TWIST score - Testicular Workup for Ischemia and Suspected Torsion score; BAL score - Boettcher Alert score

Diagnostic performance	TWIST	TT (Lim)	BAL
Sensitivity	88.2	85.3	94.1
Specificity	43.8	18.8	37.5
PPV	90.9	96.7	76.2
NPV	87.5	100	75

Overall trends

**Figure 4:**
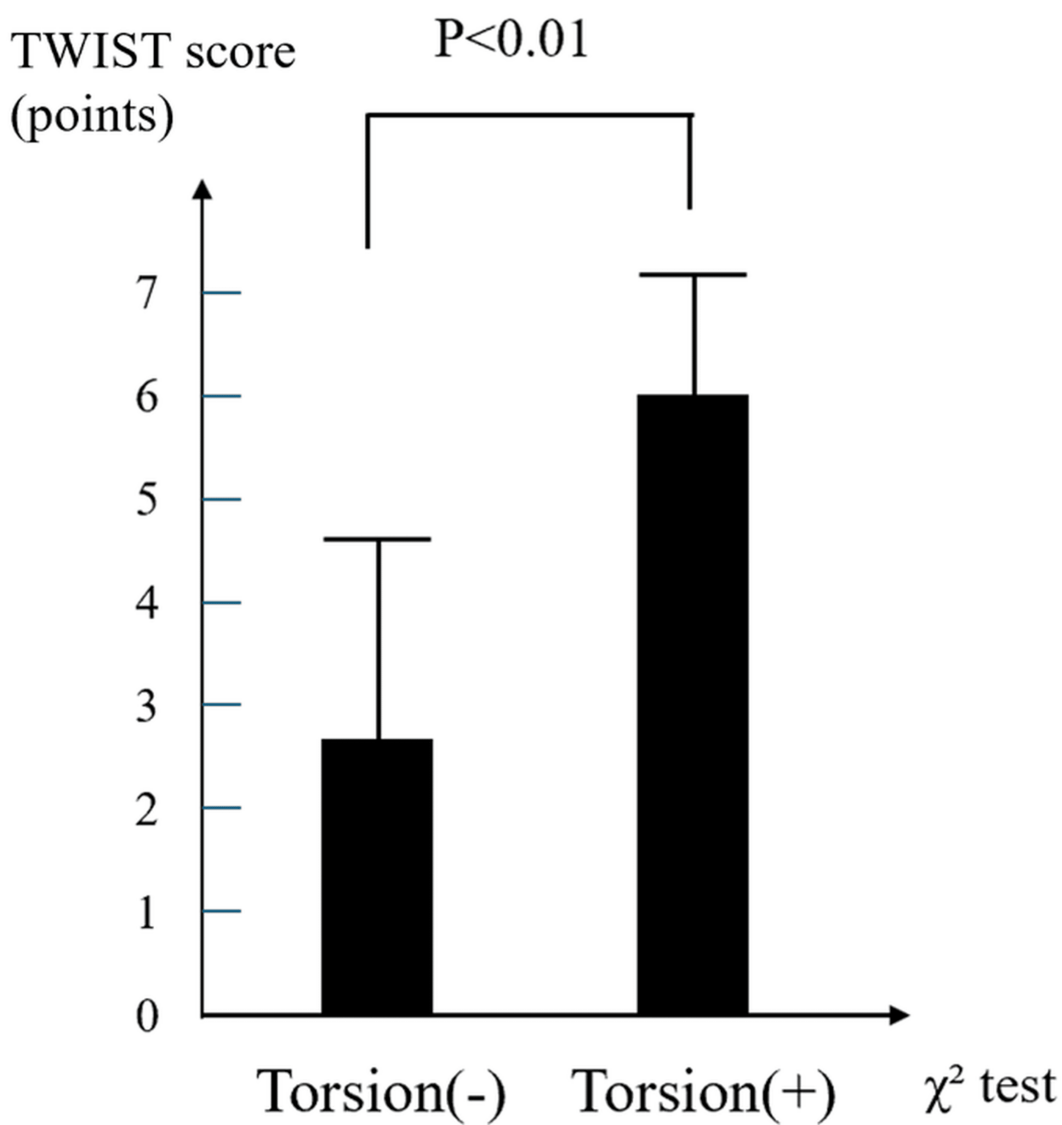
Distribution of TWIST scores by TT status The bar graph compares the mean TWIST scores of patients with and without TT. The mean TWIST score was significantly higher in the TT group than in the non-TT group (p<0.01, χ² test). TT - testicular torsion; TWIST score - Testicular Workup for Ischemia and Suspected Torsion score

**Figure 5:**
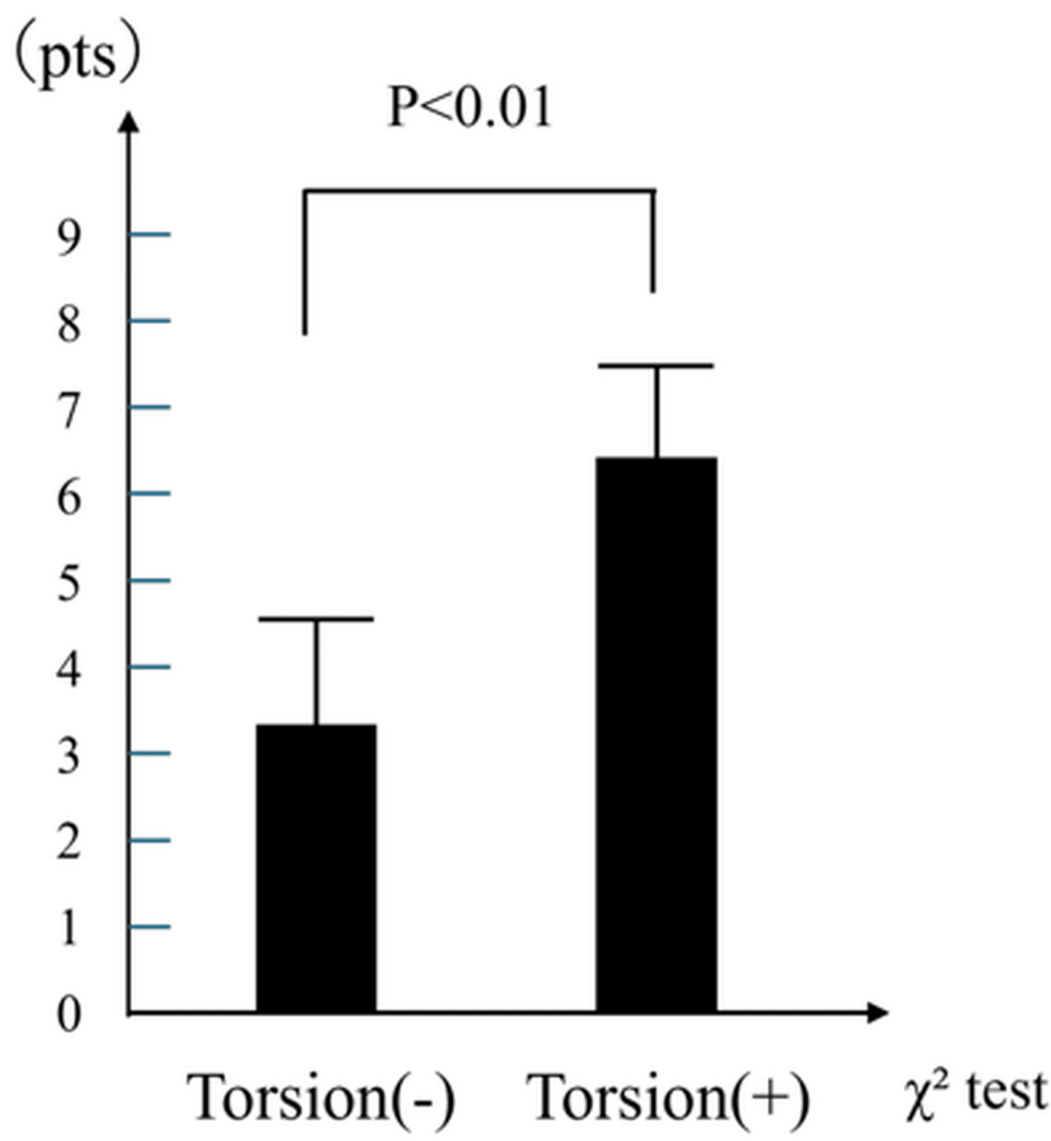
Distribution of TT (Lim) score by TT status The bar graph compares the mean TT (Lim) scores for patients with and without TT. Patients with TT had significantly higher TT (Lim) scores (6.4±1.2) compared with those without TT (3.5±1.5; p<0.01, χ² test). TT - testicular torsion

**Figure 6:**
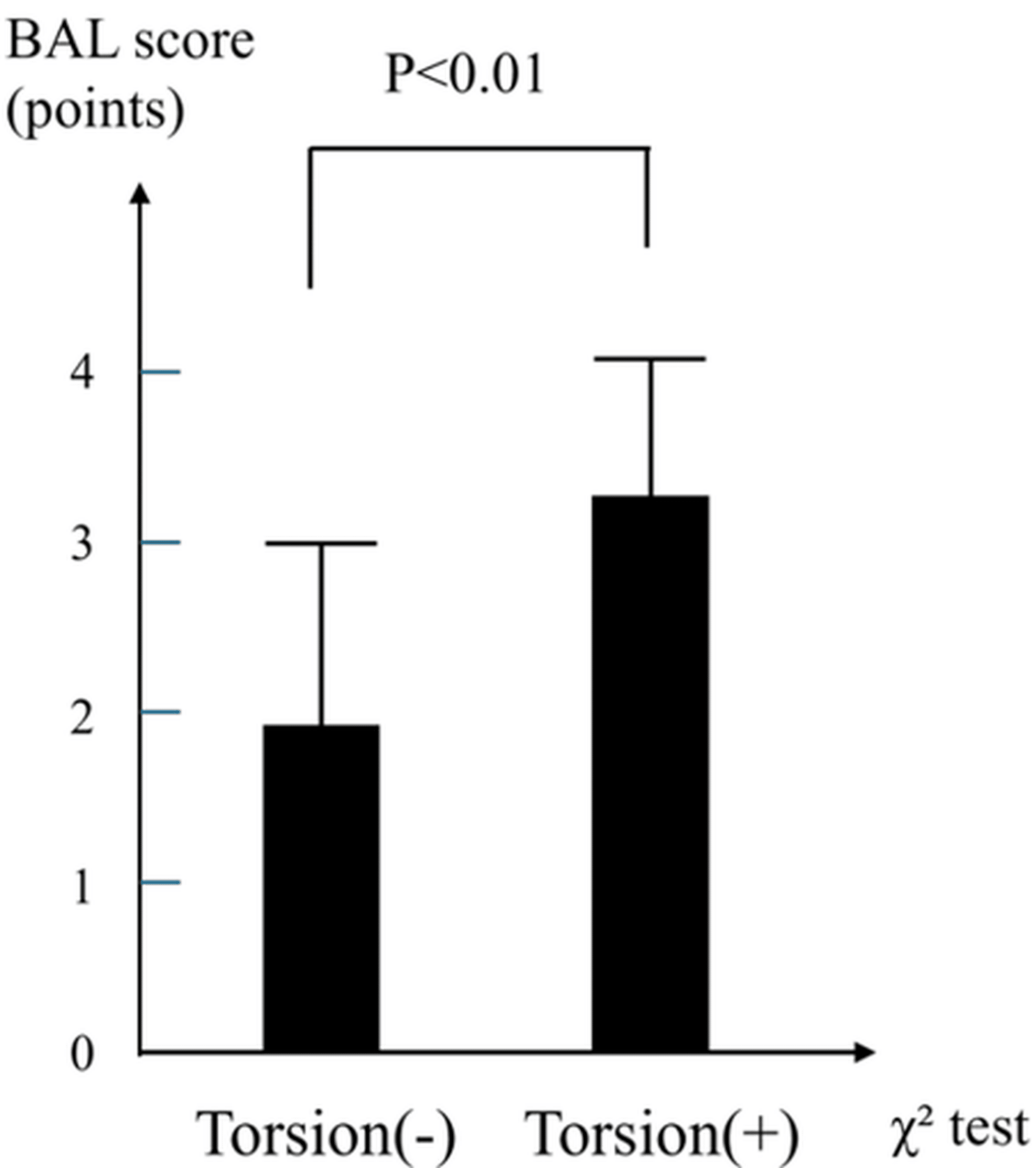
Distribution of the BAL score by TT status The bar graph compares the mean BAL scores between patients with and without TT. The mean BAL score was significantly higher in the TT group (3.3±0.8) than in the non-TT group (2.0±1.0; p<0.01, χ² test). TT - testicular torsion; BAL score - Boettcher Alert score

The summarized distributions and diagnostic accuracies are shown in Figures 4-6 and Tables 3-5, respectively. All three scoring systems effectively distinguished TT from non-TT cases, with statistically significant differences in mean scores. Among them, the TWIST and TT (Lim) scores demonstrated particularly strong discriminative performance, while the BAL score, despite its lower specificity, offered excellent sensitivity and can serve as a useful screening tool in emergency clinical settings.

**Table 3:** Cross-tabulation of TWIST score categories and TT status Among patients classified as high-risk, 30 of 33 (90.9%) had TT, indicating a strong association between higher TWIST scores and the diagnosis of TT. TT - testicular torsion; TWIST score - Testicular Workup for Ischemia and Suspected Torsion score

TWIST score	Torsion (-)	Torsion (+)	Sum
Low: 0-2	7	1	8
Intermediate: 3-4	6	3	9
High: 5-7	3	30	33
Sum	16	34	50

**Table 4:** Cross-tabulation of TT (Lim) score categories and TT status Of the 30 patients in the high-risk group, 29 had TT. All three patients in the low-risk group were non-TT cases. TT - testicular torsion

TT (Lim) score	Torsion (-)	Torsion (+)	Sum
Low: 0-1	3	0	3
Intermediate: 2-5	12	5	17
High: 6-9	1	29	30
Sum	16	34	50

**Table 5:** Cross-tabulation of BAL score categories and TT status Among 42 patients categorized as high risk, 32 had TT, while among eight patients with low risk, six were non-TT cases. These findings support the BAL score's high sensitivity (94.1%) in detecting TT, although the specificity was moderate (37.5%). TT - testicular torsion; BAL score - Boettcher Alert score

BAL score	Torsion (-)	Torsion (+)	Sum
Low: 0-1	6	2	8
High: 2-4	10	32	42
Sum	16	34	50

## Discussion

TT prevalence (68%) in our study reflects a surgically explored cohort, which differs from typical ED prevalence (15-25%). This selection and spectrum effect can inflate PPV and alter sensitivity compared with unselected ED populations. In addition, partial blinding of score abstraction may introduce retrospective confirmation bias.

This study directly compared the diagnostic accuracy of three clinical scoring systems, TWIST, TT (Lim), and BAL, in patients who underwent emergency surgery for acute scrotum. Each score demonstrated distinct characteristics, and our findings provide insight into their potential clinical applications as well as their limitations.

The TWIST score, consisting of five clinical parameters, has been extensively validated in both pediatric and adult populations. Previous meta-analyses have shown high pooled sensitivity and specificity, with an AUC exceeding 0.90 [6,10]. In our cohort, TWIST maintained high sensitivity (88.2%) and a strong PPV (90.9%), although specificity was modest at 43.8%. These findings support its value as a reliable tool for stratifying patients at high risk for torsion and for guiding surgical decision-making. Importantly, prior prospective studies have confirmed that low TWIST scores (≤2) reliably exclude torsion, while high scores (≥5) strongly predict torsion, findings that were mirrored in our analysis [7,11].

The BAL score, originally developed and validated in pediatric cohorts [9], demonstrated the highest sensitivity (94.1%) in our study, suggesting that it may be particularly useful as an initial screening tool. However, its specificity was relatively low (37.5%), resulting in a higher false-positive rate compared to TWIST or TT (Lim) scores. Although originally designed for children, the BAL score may be less accurate in adults, and its applicability in this population should therefore be interpreted with caution. Previous systematic reviews and meta-analyses have also emphasized that the diagnostic accuracy of the TWIST and BAL scores varies across age groups, with TWIST generally showing more consistent performance [6,10].

The TT (Lim) score, designed to be intuitive and usable even by non-specialists, integrates age as a diagnostic parameter along with classic clinical features. In our study, the TT (Lim) score showed a PPV of 96.7% and an NPV of 100% in the low-risk group, suggesting that it may be helpful in ruling out torsion in selected cases. However, this conclusion is limited by the very small number of patients in the low-risk category (n=3). Therefore, while promising, the TT (Lim) score requires validation in larger, prospective, and stratified cohorts before being adopted as a robust exclusion tool.

When comparing the three systems, our findings suggest complementary roles. The BAL score appears best suited for rapid screening, particularly in pediatric populations or in settings where time is critical. The TWIST score provides a more balanced diagnostic profile, making it suitable for guiding operative decisions and reducing unnecessary delays. The TT (Lim) score may have a role in ruling out torsion in low-risk patients, but its limited validation currently restricts broad applicability. Importantly, combining clinical scoring with CDUS can enhance diagnostic safety. Previous studies have reported that using TWIST in conjunction with CDUS decreases unnecessary imaging and avoids delays in surgery [11], and our findings support this integrated approach.

Another consideration is the usability of these scores by non-specialists. Previous research has demonstrated that the TWIST score has good inter-rater reliability when used by emergency physicians and non-specialists [12]. The TT and BAL scores, being simple and quick to apply, also hold promise for use in emergency settings where urologists may not be immediately available.

This study has several limitations. First, it was a retrospective, single-center study including only surgical cases, which introduced significant selection bias and limited generalizability, with a torsion prevalence (68%) much higher than in unselected emergency populations. Second, the overall sample size was relatively small, particularly in the non-TT group, which may have reduced the statistical power of subgroup analyses. Third, the wide age range (1-42 years) prevented age-stratified analysis, although the BAL score is known to be less accurate in adults. Fourth, ultrasound findings were not included, restricting the evaluation to clinical scores alone. Fifth, the low-risk TT (Lim) group was very small (n=3), which limited the robustness of its negative predictive value. Finally, inter-observer variability in the assessment of clinical signs and scoring criteria was not evaluated. These limitations should be taken into account when interpreting the results.

Despite these limitations, our results carry important clinical implications. In practice, a staged diagnostic algorithm may be considered: initial screening using BAL or TT (Lim) scores to rapidly identify high-risk or low-risk patients, followed by TWIST scoring and CDUS for definitive triage and operative decision-making. Such an approach may help shorten the time to surgical intervention, minimize unnecessary delays, and improve testicular salvage rates. Future studies should aim to validate these findings in larger, multicenter cohorts, ideally stratified by age group, and evaluate the integration of these scoring systems with bedside ultrasonography in diverse emergency settings.

## Conclusions

Each of the TWIST, BAL, and TT (Lim) scores has unique strengths and limitations, but all demonstrated clinical utility in the initial assessment of acute scrotum. The BAL and TT (Lim) scores may be particularly useful for rapid screening, whereas the TWIST score can support surgical decision-making. Prospective validation of the TT (Lim) score, as well as its integration with CDUS in clinical algorithms, is warranted.

In conclusion, this study demonstrated that the TWIST and TT (Lim) scores provide high diagnostic accuracy for identifying testicular torsion in patients undergoing surgical exploration, whereas the BAL score may be more suitable as a screening tool in pediatric populations. Each scoring system offers distinct advantages: TWIST and TT (Lim) are valuable for surgical decision-making, while BAL may help reduce missed diagnoses in clinical practice. These findings highlight the potential of integrating clinical scoring systems with CDUS to optimize diagnostic accuracy and improve testicular salvage in patients with acute scrotum. These scores remain clinically informative in surgical triage; however, estimates should be applied cautiously outside surgical cohorts, and larger prospective validations are warranted, particularly for low-risk TT (Lim) categories.
